# A multifaceted risk management program to improve the reporting rate of patient safety incidents in primary care: a cluster-randomised controlled trial

**DOI:** 10.1186/s12875-024-02476-4

**Published:** 2024-07-06

**Authors:** Marc Chaneliere, Karine Buchet-Poyau, Maud Keriel-Gascou, Muriel Rabilloud, Cyrille Colin, Carole Langlois-Jacques, Sandrine Touzet

**Affiliations:** 1 Family Medicine Department, Université Claude Bernard Lyon 1, 8 Avenue Rockefeller, Lyon, 69008 France; 2 Service Recherche Et Épidémiologie Clinique, Hospices Civils de Lyon, Pole de Santé Publique, 162 Avenue Lacassagne, Lyon, 69003 France; 3 Research On Healthcare Performance (RESHAPE), Université Claude Bernard Lyon 1, INSERM U1290, Lyon, France; 4 Service de Biostatistique Et Bioinformatique, Hospices Civils de Lyon, Pôle Santé Publique, Lyon, 69003 France; 5 UMR 5558, Laboratoire de Biométrie Et Biologie Évolutive, CNRS, Équipe Biostatistique-Santé, Villeurbanne, 69100 France

**Keywords:** Patient safety, Primary care, Risk management, Incident reporting, Cluster trial

## Abstract

**Background:**

While patient safety incident reporting is of key importance for patient safety in primary care, the reporting rate by healthcare professionals remains low. This study aimed to assess the effectiveness of a risk management program in increasing the reporting rate within multiprofessional primary care facilities.

**Methods:**

A nation-wide cluster-randomised controlled trial was performed in France, with each cluster defined as a primary care facility. The intervention included professional e-learning training, identification of a risk management advisor, and multidisciplinary meetings to address incident analysis. In the first observational period, a patient safety incident reporting system for professionals was implemented in all facilities. Then, facilities were randomised, and the program was implemented. Incidents were reported over the 15-month study period. Quasi-Poisson models were used to compare reporting rates.

**Results:**

Thirty-five facilities (intervention, n = 17; control, n = 18) were included, with 169 and 232 healthcare professionals, respectively, involved. Overall, 7 out of 17 facilities carried out the entire program (41.2%), while 6 did not hold meetings (35.3%); 48.5% of professionals logged on to the e-learning website. The relative rate of incidents reported was 2.7 (95% CI = [0.84–11.0]; p = 0.12). However, a statistically significant decrease in the incident rate between the pre-intervention and post-intervention periods was observed for the control arm (HR = 0.2; 95% CI = [0.05–0.54]; p = 0.02), but not for the intervention arm (HR = 0.54; 95% CI = [0.2–1.54]; p = 0.23).

**Conclusion:**

This program didn’t lead to a significant improvement in the patient safety incident reporting rate by professionals but seemed to sustain reporting over time. Considering that the program was fully implemented in only 41% of facilities, this highlights the difficulty of implementing such multidisciplinary programs in primary care despite its adaptation to the setting. A better understanding of how risk management is currently organized in these multiprofessional facilities is of key importance to improve patient safety in primary care.

**Trial registrations:**

The study has been registered at clinicaltrials.gov (NCT02403388) on 30 March 2015.

**Supplementary Information:**

The online version contains supplementary material available at 10.1186/s12875-024-02476-4.

## Introduction

Patient safety is defined as “the reduction of risk of unnecessary harm associated with healthcare to an acceptable minimum” [1]. Safety constitutes a key challenge for healthcare systems [2], both for hospitals and primary care, where patient safety incidents (PSIs) are a frequent occurrence. Indeed, PSI rates in primary care vary between 2 and 240 incidents per 1000 physician visits [3, 4]. In primary care, 75% of PSIs resulted in delayed or unnecessary treatments and additional costs [5]. For serious incidents, the main contributing factors (CFs) identified were human or organisational related to care [2–4].

In France, multiprofessional primary care facilities (“multidisciplinary primary care group practices ^1^”, “Primary care health centres^2^” and “primary care health poles^3^” (Appendix 1a)) have rapidly grown since the 2000s. They are expected to make an increasingly important contribution to primary care [6]. Teams in these facilities are composed of various professionals (e.g., general practitioners (GP), nurses, pharmacists, and physiotherapists…) and are involved in a shared project.

To improve patient safety in primary care, various initiatives have been suggested, including encouraging mutual support within teams, promoting a risk management policy, fostering communication with patients and their involvement, and facilitating the reporting and analysis of PSIs [7, 8]. However, while the detection and analysis of PSIs are of key importance [7], the voluntary reporting rate of professionals remains nonetheless low [3, 4]. Several barriers to reporting have been identified [9–12] and primarily include insufficient knowledge of the reporting systems, fear medical and/or legal consequences [13], and a shortage of time. To improve PSI reporting and address these obstacles, different interventions could be combined within a multifaceted risk management program, i.e., adopting specific training addressing PSIs, naming a risk management advisor for the facility and implementing methods of collective PSI analysis [7, 14–16].

The main objective of the PRisM study was to assess the effectiveness of such a multifaceted risk management program in increasing the PSI reporting rate within multiprofessional primary care facilities.

## Methods

### Study design

We conducted a nation-wide cluster-randomised trial with two parallel arms in France. A cluster was defined as a multiprofessional primary care facility (Appendix 1a). The study was conducted over two successive periods. First, an observational pre-interventional period took place over a period of 6 months to implement a PSI reporting system in all facilities. Then, a randomised interventional period took place over 15 months. In the intervention arm, PSIs continued to be collected while the risk management program was implemented. In the control arm, only PSI collection continued.

### Multiprofessional primary care facilities and participants

Facilities with at least 5 full-time equivalents (FTE) of any health professionals were eligible for enrolment in the PRisM study. All healthcare professionals were invited to participate during work hours and included GPs, nurses, pharmacists, physiotherapists, dentists, midwives, psychologists, dieticians, psychometricians, chiropodists, orthoptists, and speech therapists.

### Definition of a PSI

A PSI was defined as an “event or circumstance associated with care, that could have resulted, or did result, in harm to a patient, and which should not be repeated again” [1, 17]. PSIs refer to any event related to a patient, or any organisational issue identified by professionals as an issue to safety. For example, PSIs could be related to errors in diagnosis (wrong or delayed), investigation errors (technical acts), treatment errors (medication as nonmedication), communication or process issues (relating to medical charts), according to the TAPS taxonomy [3].

### PSI reporting system

A PSI reporting system was made available on an electronic case report form in all the facilities for the duration of the study. All participants were trained using a one-hour online session at the start of the observational period. For each PSI, it was requested that healthcare professionals report the consequences for the patient, including the degree of impairment, descriptive contextual information, contributing factors, consequences for the facility and information on corrective measures taken. Thus, professionals had the ability to submit their analysis of underlying issues and suggest improvements simultaneously or remotely from the incident report. This reporting system was successfully used in a previous French nation-wide ESPRIT study [17]. All PSIs reported were sorted by a blinded study committee. The committee was composed of one GP and the project manager trained in risk management. They verified that each event was consistent with the associated definition and was related to an activity within the facility; in the case of disagreement, a third researcher was consulted.

### Risk management program (intervention arm)

The risk management program included the following: 1) a dedicated e-learning training module for professionals; 2) the identification of a risk management advisor (RMA) within each facility; and 3) multidisciplinary meetings focusing on PSIs within each facility through experience feedback committees (EFCs) [18] or morbidity and mortality conferences (MMCs) [19, 20].

The e-learning training module addressed the identification and analysis of PSIs using a systematic approach that was carried out during multidisciplinary meetings to identify contributing factors and corrective measures [7]. A video aimed at increasing awareness so and four interactive modules were specifically developed for this program. Appendix 2 presents the content and objectives of each module. Each module was 10–15 min in duration and required each participant to complete a final self-assessment test involving clinical case scenarios and multiple-choice questions. Access to the e-learning modules was made possible throughout the duration of the intervention period.

The RMA was chosen by each facility’s team and could be any professional within the facility, regardless of age, sex, or profession. The role of the RMA was to organise and lead PSI analysis meetings, disseminate the meeting minutes among the team and regularly encourage co-workers to train and participate in the meetings.

After a minimum of 3 months of e-learning training, a cycle of six meetings, including five EFC meetings and one MMC meeting, was conducted in each facility. During each EFC meeting, participants chose a frequent but nonthreatening PSI that occurred in their facility for analysis during the next meeting. Indeed, the PSIs analysed in EFC meeting are relatively frequent near-misses or events associated with low levels of consequences for patients and the facility, as opposed to the PSIs analysed in MMR, which concern rarer events with more serious consequences. Each team named a head of analysis and a head of corrective measures. The head of analysis carried out a detailed systematic analysis of the PSI, which included the following information: a timeline; the identification of contributing factors; potential and confirmed harm to the patient, facility, or team; and proposed corrective measures. The head of corrective measures implemented and monitored the chosen measures and presented the findings during subsequent meetings. Feedback to the other professionals was provided after each EFC meeting via meeting minutes. While participants were instructed to aim for an EFC meeting of approximately one hour, the frequency was freely decided by each facility. The facilities were requested regarding the total number of meetings that were realized (from 0 to 6) and the deadline for the end of the cycle. The cycle was considered complete for 6 meetings, incomplete between 1 and 5 and non-existent if no meetings had been held. The MMC meeting took place after the EFC meetings and was conducted by healthcare professionals who presented two or three serious PSIs identified by the RMA, without preliminary analysis. All teams at each facility were invited to participate in the MMC meeting. The RMA conducted the meeting to ensure that all the professionals could freely share their point of view.

### Primary and secondary outcomes

The primary outcome was the PSI reported rate. Secondary outcomes were the nature of the PSIs according to the Threats to Australian Patient Safety (TAPS) taxonomy [3, 21], translated into French language [22]; contributing factors related to the PSIs according to the CADYA classification system ([23]; Appendix 3); PSI consequences (harm to the patient) and the nature of the consequences as outlined in the second version of the International Classification of Primary Care [24]; consequences for multidisciplinary facilities; and the corrective measures taken. All classifications were performed from data entered by professionals by the study committee who was blinded to the arms of the study.

The aspects relating to the psychometric assessment of a French version of the MOSPSC survey, the assessment of the safety culture within facilities and the qualitative analysis of the barriers to the PRisM research will be covered in separate publications.

### Data collection

Data were collected on facility characteristics, the number of professionals (and FTEs) within each facility, and characteristics regarding professionals and development of teamwork according to the facility’s organisational maturity matrix [25, 26]. PSI data were collected using the PSI reporting system. Data on the intervention implementation were collected from the e-learning audit trail and from the facilities after completing the EFC-MMC meeting cycle. All data were prospectively collected.

### Sample size

The rate of PSIs following the implementation of the reporting system without intervention was estimated at 0.3 per FTE years according to data by Zwart et al. [27]. Considering a mean of 10 FTEs per facility, a bilateral alpha risk of 5% and a coefficient of variation between facilities of 0.5, 25 facilities per arm—or 250 FTEs total—were determined necessary to detect an intervention effect with a power of 90% corresponding to a rate of PSI multiplied by 2. The number of facilities was computed with R statistical software using methodology proposed by Hans and Bennett [28].

### Randomisation

Constrained randomisation [29] was carried out to ensure the allocation of facilities was balances in the two study arms. Randomisation was stratified into quartiles based on the number of FTEs in the facilities. In each stratum, random assignment indicated by the algorithm was retained when the difference between the two arms was under a prespecified threshold regarding the mean number of the types of facilities (Appendix 1a) and the mean PSI rate measured during the pre-intervention period. Next, all possible combinations between the strata of the retained random allocations in each stratum were identified. Then, the combinations were retained when the mean differences between the two arms for each facility type and the PSI rate was under a more stringent threshold than defined in the previous step. The final randomisation list was randomly selected among the retained combinations. Constrained randomisation was implemented using the algorithm developed by Chaudhary and Moulton [30] with SAS software.

### Statistical analysis

The characteristics of the facilities and professionals were described in each arm using absolute and relative frequencies for qualitative characteristics and mean, standard deviation, and minimum and maximum values for quantitative characteristics.

The rate of reported PSIs was estimated in each facility by dividing the number of reported PSIs by the number of FTEs multiplied by the follow-up time and expressed per FTE year. The distribution of the rates was summarized in each arm as mean values and standard deviation. A quasi-Poisson model that allowed us to consider overdispersion of the rate was used to model the mean number of PSIs reported during the intervention period with an offset corresponding to the logarithm of the FTE value and quantify the effect of the intervention using a hazard ratio (HR) with a 95% confidence interval. A second quasi-Poisson model was carried out to model the mean number of reported PSIs during the pre-intervention and intervention periods. The study arm and the interaction between the period and arm was introduced in the model to quantify the effect of the intervention period on the rate of reported PSIs in both arms. The analysis was carried out on an intention-to-treat (ITT) basis and involved comparing facilities in both arms. A per-protocol analysis (PP) was also carried out to compare facilities that did not implement the risk management program to those that implemented it partially or totally. A descriptive analysis of the secondary outcomes was carried out in each arm. The analyses were performed using SAS software version 9.4 for the descriptive analysis and using R software, version 3.4.3, for the models (function glm from the package “stats”).

## Results

**Fig. 1:**
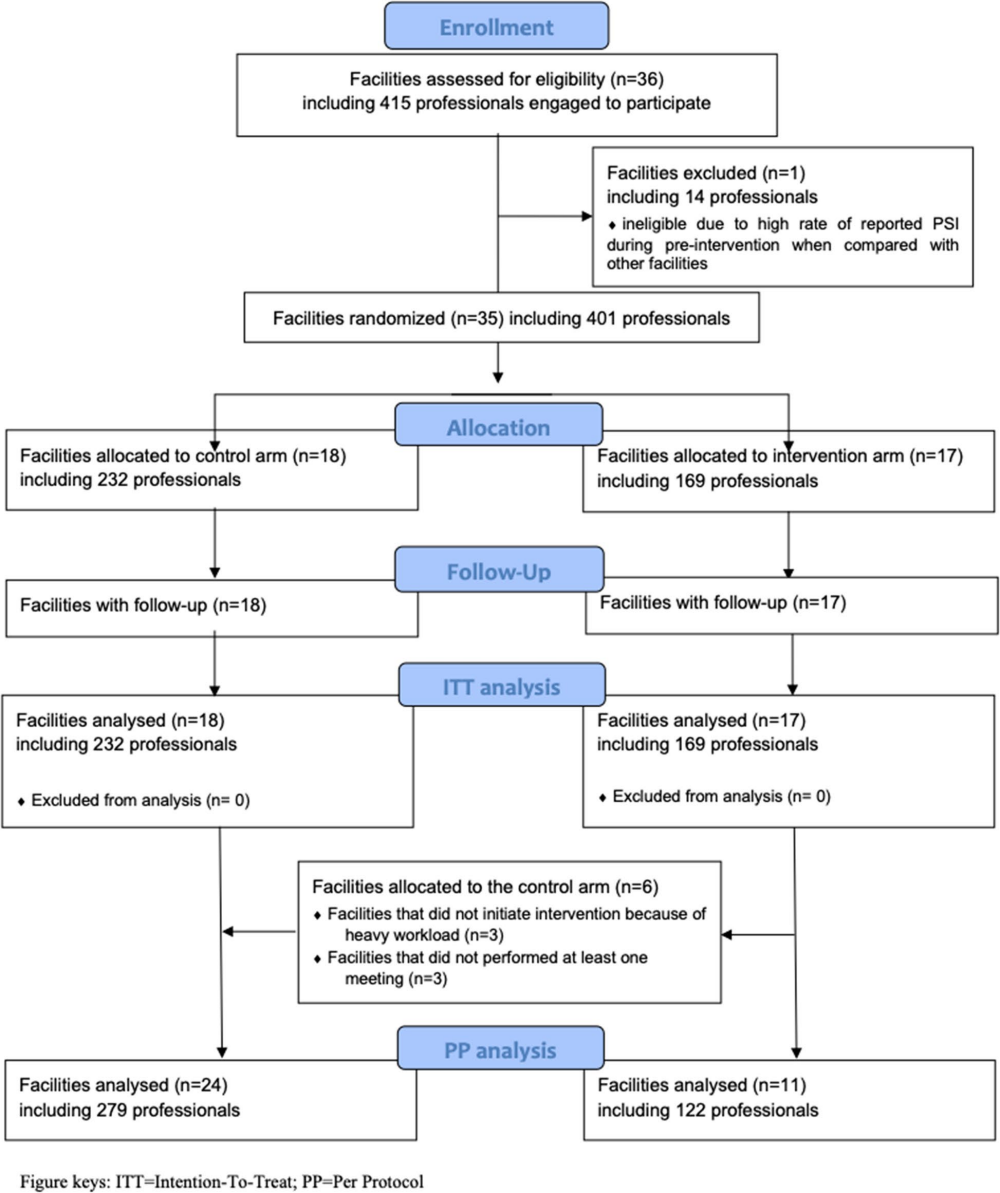
CONSORT 2010 flow diagram—facilities and professionals

Figure 1 summarises the overall design of the PRisM study according to the CONSORT 2010 Flow Diagram [31].

### Characteristics of facilities and health care professionals

Thirty-six facilities were enrolled from July 2015 to May 2017 in 9 French regions (Appendix 1b). One facility was excluded due to a high PSI rate during the pre-intervention period (12.06 per FTE year) compared with the other facilities (0.80 per FTE year; range: 0–3.8). Therefore, 35 facilities were randomised and followed for 15 months: 17 facilities (321 FTEs; mean ± SD, 18.9/facility ± 15.2) in the intervention arm and 18 (320 FTEs; 17.8/facility ± 11.7) in the control arm. Regarding the number of professionals, 169 and 232 healthcare professionals were involved in the intervention and control arms, respectively **(**Fig. 1). The characteristics of the facilities and health care professionals are shown in Table 1.

**Table 1:** Characteristics of facilities and health care professionals

	**Control**	**Intervention**
**Facilities**	***n* = 18**	***n* = 17**
Facility type		
* - Multidisciplinary primary care group practices*, n (%)	10 (55.6)	9 (52.9)
* - Primary care health pole*, n (%)	4 (22.2)	3 (17.7)
* - Primary care health centre*, n (%)	4 (22.2)	5 (29.4)
FTE of healthcare professionals, n	320.3	321.1
FTE of healthcare professionals per facility, mean (SD); Min–Max	17.8 (11.7); 5–46	18.9 (15.2); 5–64
Risk management system within facility, n (%)	4 (22.2)	2 (11.8)
Development of teamwork (according to facility organisational maturity matrix)		
* - Practice meetings within facility, n (%)*	4 (22.2)	4 (23.5)
* - Use of guidelines, n (%)*	3 (16.7)	1 (5.9)
* - Care coordination, n (%)*	3 (16.7)	6 (35.3)
* - Teamwork dynamic, n (%)*	4 (22.2)	6 (35.3)
PSI/FTE/year, mean value (SD); Min–Max	0.80 (0.89); 0–3.43	0.81 (0.80); 0–3.16
**Health care professionals**	***n* = 232**	***n* = 169**
Age (in years), mean (SD); Min–Max	46.6 (11.7); 24–68	45.9 (11.0); 28–68
Women, n (%)	159 (68.5)	123 (72.8)
Duration of activity within facility (in years), mean (SD); Min–Max	6.0 (7.4); 1–36	6.8 (7.7); 1–36
Previous risk management training, n (%)	5 (2.2)	4 (2.4)
Healthcare category		
* - General practitioner, n (%)*	78 (33.6)	70 (41.4)
* - Nurse, n (%)*	55 (23.7)	46 (27.2)
* - Physiotherapist, n (%)*	19 (8.2)	12 (7.1)
* - Other, n (%)*	80 (34.5)	41 (24.3)

*Keys*: *PSI* Patient safety incident, *SD* Standard deviation, *FTE* Full-time equivalent

### Characteristics of PSIs in the pre-intervention observational period

**Fig. 2:**
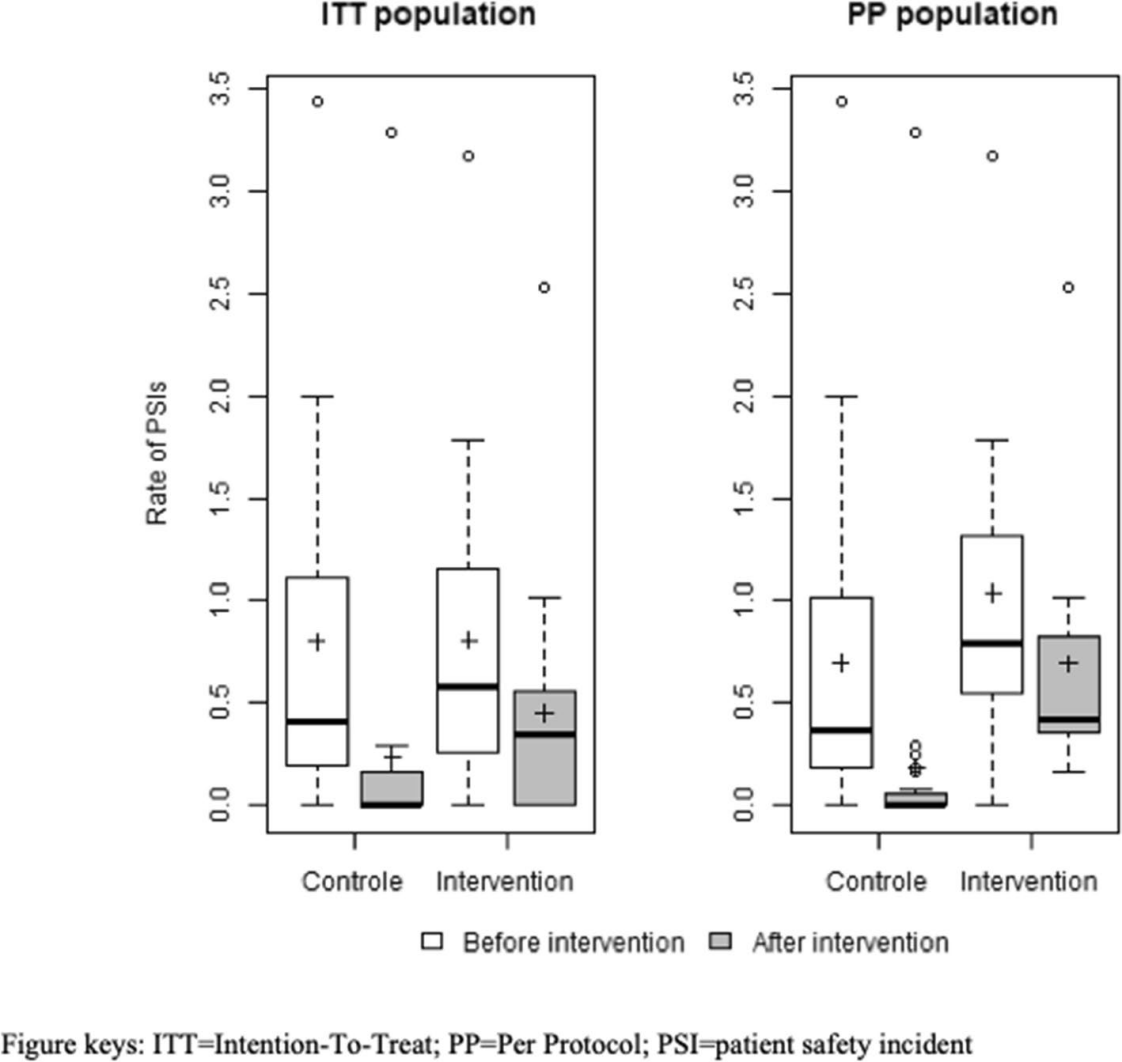
Boxplot of PSI rate evolution in both arms in ITT and PP populations

Among PSIs reported during pre-intervention, 97.4% (114/117) and 99.2% (116/117) were validated for the intervention and control arms, respectively. The mean rate of PSIs per FTE year was 0.81 (SD = 0.80) in the intervention arm and 0.80 (SD = 0.89) in the control arm (Fig. 2). PSI characteristics are shown in Table 2. More than 80% of PSIs in each arm represented single patient incidents (i.e., related to one patient). PSI typologies were mainly related to errors in practice and in the healthcare system (intervention: 36% vs control: 34.5%) and to medication errors (21.0% vs 30.2%). Contributing factors according to the CADYA classification were identified for each PSI (1.8 (± 1.2) vs 1.4 (± 1.2)). The main contributing factor related to process of care or human factors (57.9% vs 59.5%). Less than one-third of PSIs had an established harmful effect on patient health, mainly without serious harm, remained at a general level (i.e., health maintenance/prevention), and was associated with a delay in care. Professionals identified approximately one-quarter of PSIs with potential consequences at the facility level, which mainly involved a loss in patient confidence and failure in the facility Information System. Corrective measures were proposed for 89% of PSIs, with 70.5% vs 65.3% of measures related to the organisation within the facility or to medical training and care practice.

**Table 2:** Characteristics of PSIs in the observational pre-intervention period

**Characteristics**	**Control** *n* = 116	**Intervention** *n* = 114
Single patient PSI^a^, n (%)	101 (87.1)	92 (80.7)
**PSI types (according to the TAPS study taxonomy)**		
* - Errors in practice and healthcare system, n (%)*	40 (34.5)	41 (36.0)
* - Investigation errors, n (%)*	4 (3.4)	13 (11.4)
* - Medication errors, n (%)*	35 (30.2)	24 (21.0)
* - Treatment errors (nonmedication), n (%)*	9 (7.8)	3 (2.6)
* - Communication errors and process errors not otherwise specified, n (%)*	11 (9.5)	13 (11.4)
* - Errors in diagnosis, n (%)*	4 (3.4)	5 (4.4)
* - Errors in managing patient care, n (%)*	13 (11.2)	15 (13.2)
**PSI contributing factors**	*n* = 116	*n* = 114
Contributing factors, mean (SD); Min–Max	1.4 (1.2); 0–4	1.8 (1.2); 0–5
**Type of contributing factors, according to CADYA**		
* - Process of care, n (%)*	40 (34.5)	36 (31.6)
* - Human factors, n (%)*	29 (25.0)	30 (26.3)
* - Technical factors, n (%)*	9 (7.8)	19 (16.7)
* - Environmental factors, n (%)*	22 (19.0)	16 (14.0)
* - Others, n (%)*	16 (13.8)	13 (11.4)
**PSI consequences**		
***Consequences for patients***	*n *= 116	*n* = 112
PSIs leading to a harmful effect^b^ on patient's health, n (%)	36 (31.0)	22 (19.6)
PSIs leading to death, life-threatening PSIs, or permanent body impairment, n (%)	4 (11.2)	4 (18.2)
**Type of consequences for patients, according to the ICPC**		
* - A98-Health maintenance/prevention, n (%)*	7 (19.4)	5 (22.7)
* - A85-Adverse effect medical agent, n (%)*	9 (25.0)	0
* - A87-Complication of medical treatment, n (%)*	4 (11.1)	2 (9.1)
* - Others from A -General and unspecified category, (n%)*	3 (8.3)	2 (9.1)
* - Others, n (%)*	13 (36.1)	13 (59.1)
***Consequences for facilities***	*n* = 116	*n* = 114
Consequences per PSI, mean (SD); Min–Max	0.31 (0.60); 0–3	0.27 (0.57); 0–3
PSIs with at least one consequence, n (%)	29 (25.0)	25 (21.9)
PSIs with at least one true^c^ consequence, n (%)	8 (6.9)	11 (9.6)
PSIs with at least one potential consequence, n (%)	26 (22.4)	19 (16.7)
**Type of consequences for facilities**		
* - Loss in patient’s confidence in the facility and providers, n (%)*	8 (29.6)	2 (9.1)
* - Failure in the facility's information system, n (%)*	3 (11.1)	5 (22.7)
* - Low level of safety in the facility, n (%)*	4 (14.8)	1 (4.5)
* - Disruption to healthcare professional’s schedule, (%)*	2 (7.4)	3 (13.6)
* - Others, n (%)*	10 (37)	11 (50)
**Corrective measures for PSIs**	*n* = 116	*n* = 111
Corrective measures per PSI, mean (SD); Min–Max	1.1 (0.6); 0–3	1.3 (0.8); 0–4
PSIs that led to at least one corrective measure, n (%)	104 (89.7)	99 (89.2)
**Type of corrective measures**^**d**^		
* - Measures related to the medical training and care practice*, n (%)	37 (29.8)	28 (20.1)
* - Measures related to material and technical level within the facility*, n (%)	12 (9.7)	15 (10.8)
* - Measures related to the organization within the facility*, n (%)	44 (35.5)	70 (50.4)
* - Measures related to the management of human factors*, n (%)	10 (8.1)	8 (5.8)
* - Other*, n (%)	21 (16.9)	18 (12.9)

*Abbreviations*: *PSI* Patient safety incident, *SD* Standard deviation, *No.* Number, *ICPC* International classification of primary care safety, *CADYA* Categorization of errors in primary care, *TAPS* threats to Australian patient safety

^a^Refers to a PSI related to a unique patient

^b^Refers to all unnecessary harm to a patient (potential as well as real)

^c^Refers to PSIs with a real reported consequence at the level of the facility

^d^It was possible to have more than one corrective measure per PSI; percentage values represent the total number of measures identified

### Implementation of the risk management program in the intervention arm

Among the 169 professionals in the intervention arm, 48.5% (*n* = 82) logged on to the e-learning website and looked at the introductory video on PSI identification (Appendix 2). The first generic module was validated by 39.1% (*n* = 66) of the professionals, whereas 11.2% (*n* = 19) validated module 4, which was specific to the RMA. The EFC-MMR meeting cycles were carried out completely in 7 facilities (41.2%), incompletely in 4 facilities (23.5%), and not at all in 6 facilities (35.3%). A mean of 81% of professionals across all facilities that completed the entire cycle logged on to the e-learning website. In contrast, 37% of professionals among facilities that partially completed the cycle, and 3% of professionals from 6 facilities that did not hold meetings, logged on to the e-learning website. The time between randomisation and the occurrence of the first meeting was 3.3 months ± 2.1, and the time between the first meeting to the 6th meeting was 7.7 months ± 2.2 for facilities that completed the entire cycle. Characteristics of the implemented risk management program are presented in Table 3.

**Table 3:** Implementation of the PRisM risk management program in the intervention arm

**Training by e-learning at participant level**	***n* = 169**
Participants logged on to video on PSI identification, n (%)	82 (48.5)
Time (in days) between when training was made available and first connection, mean (SD)	38 (35)
Participants that completed e-learning modules:	
- Interactive module 1: What are EFC and MMC meetings, n (%)	66 (39.1)
- Interactive module 2: How to investigate a PSI, n (%)	36 (21.3)
- Interactive module 3: How to manage corrective actions, n (%)	34 (20.1)
- Interactive module 4: How to run EFC and MMC meetings, n (%)	19 (11.2)
**Training by e-learning at RMA level**	***n* = 17**
RMA logged on to video on PSI identification, n (%)	11 (64.7)
Time (in days) between when training was made available and the connection, mean (SD)	12 (12)
**Training by e-learning at facility level**	***n* = 17**
Ratio (%) of participants logged on by the overall facilities, mean (SD); min–max	43 (38); 0–100
Ratio (%) of participants logged on by facilities:	
- carried out a complete EFC-MMC cycle^a^, mean (SD); min–max (*n* = 7)	81 (16); 58–100
- carried out an incomplete EFC-MMC cycle^a^, mean (SD); min–max (*n* = 4)	37 (16); 27–60
- did not perform a least one meeting (EFC/MMC), mean (SD); min–max (*n* = 6)	3 (7); 0–17
Time (in days) between the date when training was made available and the first connection for facilities:	
- carried out a complete EFC-MMC cycle^a^, mean (SD) (*n* = 7)	38 (14)
- carried out an incomplete EFC-MMC cycle^a^, mean (SD) (*n* = 4)	55 (31)
- did not perform a least one meeting, mean (SD) (*n* = 6)	-
**EFC-MMC meeting cycle at facility level**	***n* = 17**
Facilities that carried out a complete EFC-MMC cycle^a^, n (%)	7 (41.2)
Facilities that carried out an incomplete EFC-MMC cycle^a^, n (%)	4 (23.5)
Facilities that did not conduct at least one meeting, n (%)	6 (35.3)
EFC–MMC meeting number, mean per facility (SD); min–max	3.2 (2.8); 0–6
Time (in months) between first and 6th meetings for facilities that carried out a complete EFC-MMC meeting cycle, mean (SD) (*n* = 7)	7.7 (2.2)

*Abbreviations*: *SD* Standard deviation, *EFC* Experience feedback committee, *MMC* Morbidity and mortality conference, *RMA* Risk management advisor

^a^Five EFCs meetings and one MMC meeting were required to complete an EFC-MMC cycle

### Impact of the risk management program on PSI rate

In the ITT analysis among PSIs reported during the intervention period, 91.5% (162/177) were validated in the intervention arm and 90.9% (60/66) in the control arm. The characteristics of the PSIs reported by professionals during the intervention period were similar to those reported in the pre-intervention period (Table 4).

**Table 4:** Characteristics of PSIs after 15 months of follow-up

**Characteristics**	**Control** *n* = 60	**Intervention** *n* = 162
Single patient PSI^a^, n (%)	47 (78.3)	136 (84.0)
**PSI types (according to the TAPS study taxonomy **		
* - Errors in practice and healthcare system, n (%)*	18 (30.0)	51 (31.5)
* - Investigation errors, n (%)*	12 (20.0)	18 (11.1)
* - Medication errors, n (%)*	10 (16.7)	44 (27.2)
* - Treatment errors (nonmedication), n (%)*	4 (6.7)	5 (3.1)
* - Communication errors and process errors not otherwise specified, n (%)*	8 (13.3)	21 (13.0)
* - Errors in diagnosis, n (%)*	3 (5.0)	7 (4.3)
* - Errors in managing patient care, n (%)*	3 (5.0)	14 (8.6)
**PSI contributing factors**	*n* = 60	*n* = 162
Contributing factors, mean (SD); Min–Max	1.7 (1.3); 0–5	2.1 (1.2); 0–5
**Type of contributing factors, according to CADYA**		
* - Process of care, n (%)*	13 (21.7)	40 (24.7)
* - Human factors, n (%)*	20 (33.3)	47 (29.0)
* - Technical factors, n (%)*	7 (11.7)	29 (17.9)
* - Environmental factors, n (%)*	9 (15.0)	32 (19.8)
- Others, n (%)	11 (18.3)	14 (8.6)
**PSI consequences**		
*** Consequences for patients***	*n* = 60	*n* = 162
PSIs leading to a harmful effect^b^ on patient's health, n (%)	8 (13.3)	41 (25.3)
PSIs leading to death, life-threatening PSIs, or permanent body impairment, n (%)	2 (25.0)	10 (24.3)
**Type of consequences for patients, according to the ICPC**		
* - A: General and unspecified (including mainly A98-Health maintenance/prevention), n (%)* * - S: Skin (including mainly S20—skin injury other), n (%)*	3 (37.5) 1 (12.5)	6 (16.7) 6 (16.7)
* - K: Cardiovascular*	0	7 (19.4)
* - Others, n (%)*	4 (50)	17 (47.2)
*** Consequences for facilities***	*n* = 58	*n* = 160
Consequences per PSI, mean (SD); Min–Max	0.5 (0.8); 0–3	0.3 (0.8); 0–3
PSIs with at least one consequence, n (%)	20 (33.3)	41 (25.3)
PSIs with at least one true^c^ consequence, n (%)	10 (16.7)	9 (5.6)
PSIs with at least one potential consequence, n (%)	15 (25.0)	37 (22.8)
**Type of consequences for facilities**		
* - Loss in patient’s confidence in the facility and providers, n (%)*	5 (21.8)	10 (23.8)
* - A change in the organizational structure of the facility (procedure), n (%)*	3 (13.0)	5 (11.9)
* - Failure in the facility's Information System, n (%)*	3 (13.0)	3 (7.1)
*- Low level of safety in the facility, n (%)*	0	5 (11.9)
* - Disruption to healthcare professional’s schedules, (%)*	4 (17.4)	1 (2.5)
* - Loss of patient base, (%)*	2 (8.7)	3 (7.1)
* - Others, n (%)*	6 (26.1)	15 (35.7)
**Corrective measures for PSIs**	*n* = 58	*n* = 160
Corrective measures per PSI, mean (SD); Min–Max	1.2 (0.6) (0–3)	1.3 (0.7) (0–4)
PSIs that led to at least one corrective measure, n (%)	54 (93.1)	154 (96.3)
**Type of corrective measures**^d^		
* - Measures related to the medical training and care practice*, n (%)	26 (37.7)	77 (36.3)
* - Measures related to material and technical level within the facility*, n (%)	6 (8.7)	24 (11.3)
* - Measures related to the organization within the facility*, n (%)	18 (26.1)	71 (33.5)
* - Measures related to the management of human factors*, n (%)	12 (17.4)	19 (9.0)
* - Other*, n (%)	7 (10.1)	21 (9.9)

*Abbreviations*: *PSI* Patient safety incident, *SD* Standard deviation, *No.* Number, *ICPC* International Classification of Primary Care, *CADYA* Categorization of errors in primary care, *TAPS* Threats to Australian Patient Safety

^a^Refers to a PSI related to a unique patient

^b^Refers to all unnecessary harm to a patient (potential as well as real)

^c^Refers to PSIs with a real reported consequence at the level of the facility

^d^It was possible to have more than one corrective measure per PSI; percentage values represent the total number of measures identified

The mean rate of reported PSIs was 0.45 ± 0.63 per FTE year in the intervention arm vs 0.24 ± 0.77 FTE year in the control arm (Fig. 2). The HR of PSIs in the intervention vs control arms was 2.7 (95% CI = [0.63–17.3], *p* = 0.23) and remained non-significant in the second model that included the pre-intervention period (HR = 2.7, 95% CI = [0.84–11.0], *p* = 0.12). The HR estimate in the second model for the intervention vs pre-intervention periods in the control arm was 0.2 (95% CI = [0.05–0.54], *p* = 0.02), indicating a statistically significant decrease in the PSI rate between the pre-intervention and intervention periods. Conversely, a decrease in the PSI rate for the intervention arm was not statistically significant (HR = 0.54, 95% CI = [0.2,1.54], *p* = 0.23).

In the per-protocol analysis (Fig. 2), the effect of the intervention on the PSI rate was not statistically significant (HR = 4.34, 95% CI = [0.95–30.25], p = 0.08). In the second model including the pre-intervention period, the effect of the intervention reached statistical significance (HR = 4.35, 95% CI = [1.31–18.62], p = 0.028). The HR for the intervention period vs pre-intervention period in the control arm was estimated at 0.17 (95% CI = [0.04–0.61], *p* = 0.011), indicating a statistically significant decrease in the PSI rate, whereas a decrease in the PSI rate between both periods was not statistically significant in the intervention arm (HR = 0.63, 95% CI = [0.22–1.98], *p* = 0.4).

## Discussion

### Summary of main outcomes

The PRisM program did not increase the healthcare professional reporting rate of PSIs per FTE years; however, there was significant variability between facilities concerning the implementation of the program. While all facilities designated their own RMA, only 7 out of 17 facilities carried out the entire EFC-MMC cycle and had > 80% of healthcare professionals complete trained by e-learning, while 4 facilities partially completed the cycle, with approximately 40% of professionals completing training. The PP analysis with these 11 facilities showed a statistically significant effect of the program on PSI rate in the model that included the pre-intervention period, which suggests that the PRisM program promotes sustained PSI reporting over time but does not improve PSI reporting by healthcare professionals. Overall, 230 and 222 PSIs were reported in the pre-intervention and intervention periods, respectively, and were frequently related to errors in practice and the healthcare system or errors related to medication. There were few severe adverse patient outcomes, with the main contributing factors relating to care processes or human factors. Consequences regarding facilities mainly involved a loss in patient confidence and changes at the organisational level. Corrective measures were identified more frequently related to the organisation within the facility or the medical training and care practice. Thus, our work improves understanding of the nature of the most frequent and serious PSIs in primary care, the associated contributing factors and, more remarkably, the corrective measures implemented.

### Comparison with existing literature

It is essential to promote the implementation of risk management systems in facilities given their increasingly important role in primary care [32, 33]. To achieve this goal, it would appear advisable to design an intervention that would combine several elements recommended for improving patient safety in primary care [8]. In the PRisM study, the PSI rate, following the implementation of a reporting system without additional interventions, reaches 0.8 per Full-Time Equivalent (FTE) year, in the pre-intervention period in contrast to about 0.2–0.3 estimated from other studies [5, 27, 34]. The decreased rate of reported PSIs over time needs comment, as it was observed in both arms. As suggested by the relatively high basal PSI rate in the PRisM study, we postulate an overestimation in the pre-interventional period and weariness among professionals to report over time, rather than a decrease in reported PSIs linked to the implementation of efficient corrective measures (which would be more important in the intervention arm). As previously suggested [12], the declining trend in reporting could also be explained by the greater involvement of facilities at the beginning, due to urgent safety issues to be reported for processing. Once resolved, they may reduce their level of involvement. Moreover, assignment to the control arm may have demotivated some teams from participating [34], despite access to the program at the end of the study. However, the intervention was observed to sustain a certain basal level of PSI reporting with a post-intervention PSI rate of 0.45 per FTE year vs 0.24 in the control arm, more comparable to other [5, 27, 34]. The literature consistently highlights an underreporting of PSI by professionals [35], even in case of an intervention dedicated to support patient safety culture in primary care [34]. In addition, in the PRisM study the standard deviation of the PSI rate was high, similar to the result itself. In our opinion, this reflects a wide disparity between facilities when it comes to reporting incidents, as it was previously observed [12]. PSIs related to errors in the organisation of the healthcare system, or related to medications were most frequent reported, an observation that has been identified previously [3, 4, 36]. In response, considering alternative strategies, such as trigger tools emerges as a promising avenue. The contributing factors identified according to CADYA [22, 23] are consistent with data from a previous French national study [17, 23], in which almost a third of CFs were related to human factors. The PRisM study also enabled us to explore the development of corrective measures by healthcare professionals. In nearly 90% of incidents, healthcare professionals proposed at least one measure, which is remarkable. The fact that these corrective measures concerned training and organisation within the facilities, representing nearly 2/3 of PSIs, indicates that it is possible to promote an integrated approach to improving the quality and safety of care.

### Strengths and limitations

The primary outcome of this study was the reporting rate of PSIs per FTE year, which is one of the most widely used patient safety indicators. Others outcome measures, such as the safety climate or data on patient morbidity-mortality, may have been more suitable. However, to assess the safety climate remains difficult and data on morbidity-mortality could suffers from a relatively low frequency of severe incidents observed in primary care [17], which raises the question of their representativeness. Regarding the choice of taxonomy used for PSI analysis, the WHO International Classification for Patient Safety allows for coding all elements of systemic analysis (dysfunctions, consequences, etc.) [37]. As it seems to be more complex to use, we have opted for a method that separates out all the elements; for instance, the TAPS taxonomy for the nature of PSI, considering its international applicability (facilitating comparisons) [3, 21]. Additionally, for the classification of dysfunctions, we employed the CADYA classification [23] supported by the French High Health Authority for the analysis of incidents in primary care.

The PRisM intervention aimed to provide key components of an integrated risk management system in a primary care facility. Multiprofessional facilities is a recent modality in France and, therefore, we focused on healthcare professionals. The patients were not explicitly encouraged to report incidents. However, several incident reports stemmed from patient detection, which was subsequently reported to the healthcare professional. Patient involvement should be considered in future studies, especially in PSI reporting as it constitutes an underutilized source in primary care.

Teamwork itself influences the implementation of a risk management program [38] as much as it can itself benefit from it [39]. Although a limitation of this work is that teamwork was not specifically addressed by the study program, the e-learning modules provided instructions on how to run a multiprofessional meeting and the EFC-MMC cycle strongly involved a teamwork dimension. The mean duration of activity varied among professionals and the existence of strong and long-standing team dynamics as a prerequisite for successful intervention has not been explored [40]. Barriers to PSI reporting by healthcare professionals have been identified [41]; however, these are not specific to primary care. Barriers such as the fear of legal consequences may not have been sufficiently addressed in the e-learning module implemented in this study. To assess the reporting rate of PSIs, a cluster-randomised controlled trial was preferred to a stepped wedge randomised controlled trial design [42] because the risk of contamination bias was small, given the distribution of facilities across the country. Although a stepped wedge design would have provided the intervention to all facilities, the full program was freely available to all facilities after the study. Prior to the study, we postulate the risk that the facilities would be very heterogeneous and that it could be difficult to implement the program. The benefits of including a PP analysis (to explore the effect of truly receiving the PRisM program) was considered, in addition to the main ITT analysis. The main limitation of this study remains the lack of statistical power as 35 facilities were randomised instead of 50 that were initially intended, which highlights the difficulty of enrolling professionals to participate in research in primary care in France [43]. Indeed, with 17 facilities enrolled per arm instead of 25, the power to conclude to a difference between the two groups is 76% instead of 90% expected, even if the number of enrolled FTEs exceeds the expected 500, due to several facilities with over 50 professionals. Furthermore, the facilities were located throughout the country and were, therefore, representative of multiprofessional primary care facility organisations in France. Moreover, while GPs constitute the majority of healthcare professionals, a strength of this study is that a diverse range of professionals, including nurses and, to a lesser degree, physiotherapists, were well represented. Indeed, PSI reporting is known to be enhanced in facilities comprising several types of professionals [12].

### Implications for research and/or practice

The observation that the reporting of PSIs by professionals continued in the intervention group suggests the program had a positive effect on reporting dynamic. Thus, the reporting dynamic could be sustainable, subject to a minimum of consideration for patient safety in teamwork. However, as observed in other study [34] program implementation varied significantly between facilities, emphasizing that teamwork dynamic within a facility is a key factor [44]. Generic barriers and facilitators have already been identified in the literature [45], but they are not specific for primary care. A qualitative study led by a sociologist was conducted to assess the barriers and facilitators to the PRisM program among primary care facilities. Several aspects of the program seemed to have worked well, such as the identification of an RMA, which was effective in all facilities. This RMA was, in many cases, the person who had the greatest leadership over the team. It would seem appropriate to rely on this person each time in the context of a quality and safety approach [46], but this was not always sufficient. This finding is consistent with other studies suggesting that strong leadership devoted to patient safety constitutes a key factor in developing a favourable patient safety climate [47]. Our future work will aim to identify the organisational and leadership arrangements in facilities that are most likely to result in the successful implementation of risk management programs.

Although the PSI reporting system was designed to be ergonomic, we can probably assume that the time associated with collecting data, in addition to the existing information system, constituted a barrier after initial enthusiasm for the program waned. It would, therefore, seem necessary to consider the possibility of integrating PSI reporting through the medical chart system and/or facilitating automatic data extraction. As suggested by the results of the qualitative study conducted at the end of our study, we propose the integration of a simple checkbox to indicate a potential PSI in the medical chart to subsequently facilitate their exploitation. Logistical support (i.e., regarding the analysis of documents or PSI reporting system use) is a key element for a successful program. In France, such methodological support is already offered to teams in hospitals through dedicated services. Durable support should also be provided through regional support structures for primary care facilities. As a related issue, there is currently no specific indicator related to patient safety in the maturity matrix of primary care structures in France [25]. As a result, the assessment of patient safety remains formally underdeveloped, primarily relying on indicators of medication-related harm, focusing more on prescribing professionals than on the facilities themselves.

Regarding the modalities of analysis in the structures, EFC or MMC meetings appear as viable solutions as they may contribute fostering a culture of reporting PSIs since incidents are processed by the team itself [15, 47]. These meetings should be strongly promoted by public authorities via specific funding for the facilities implementing them. The collection of improvement measures related to PSI occurrence and their dissemination to other structures may represent an opportunity.

## Conclusion

The aim of the PRisM study was to assess the effectiveness of a risk management program in increasing the patient safety incidents reporting rate within multiprofessional primary care facilities. Thus, a multi-faceted intervention combining several elements has been implemented in France through a nationwide cluster-randomised controlled trial. The program didn’t lead to a significant improvement in the patient safety incident reporting rate by professionals, but it seemed to sustain reporting over time. A more profound understanding of how risk management is currently organized in these multiprofessional facilities remains of key importance to improve patient safety in primary care. Thus, the procedures for risk management could be based on less formal practices at the primary care team level. Patient involvement should also be promoted in incident reporting.

### Supplementary Information

Supplementary Material 1.

Supplementary Material 2.

Supplementary Material 3.

## Data Availability

The study protocol and data are available upon reasonable request.

## Abbreviations

CADYA: CAtégorisation des DYsfonctionnements en Ambulatoire (or Categorization of Errors in Primary Care)
CF: Contributing factor
EFC: Experience feedback committee
FTE: Full-time equivalents
GP: General practitioners
HR: Hazard ratio
ITT: Intention-to-treat
MMR: Morbidity and mortality conference
PP: Per-protocol
PSI: Patient safety incident
RMA: Risk management advisor
TAPS: Threats to Australian Patient Safety
