# Supplementary material for: A multifaceted risk management program to improve the reporting rate of patient safety incidents in primary care: a cluster-randomised controlled trial
Source: BMC Prim Care. 2024 Jul 6;25:244. doi: 10.1186/s12875-024-02476-4 (PMC11227140; doi:10.1186/s12875-024-02476-4)

**Appendix 1a** (notes to international readers)

*^1^ Multidisciplinary primary care group practices (MPCGP) for Maisons de santé pluridisciplinaires* refers to medical group practices in which medical and paramedical private caregivers provide primary care service.

*^2^ Primary care health centres (PCHC) for Centre de santé* refers to outpatient medical care centres staffed with medical and paramedical professionals who provide generalist and specialist care. They are supported by private associations, insurance companies or townships.

*^3^ Primary care health poles (PCHP) for Pôle de santé* refers to an administrative office whose task is to coordinate healthcare between private practitioners from both the medical and paramedical levels in a territory. The primary duties consist of removing duplicate activities and promoting the shared or common use of facilities and equipment.

In each case (1,2,3), the caregivers were involved in a common health project.

**Appendix 1b (map of France with localities and various types of facilities)**


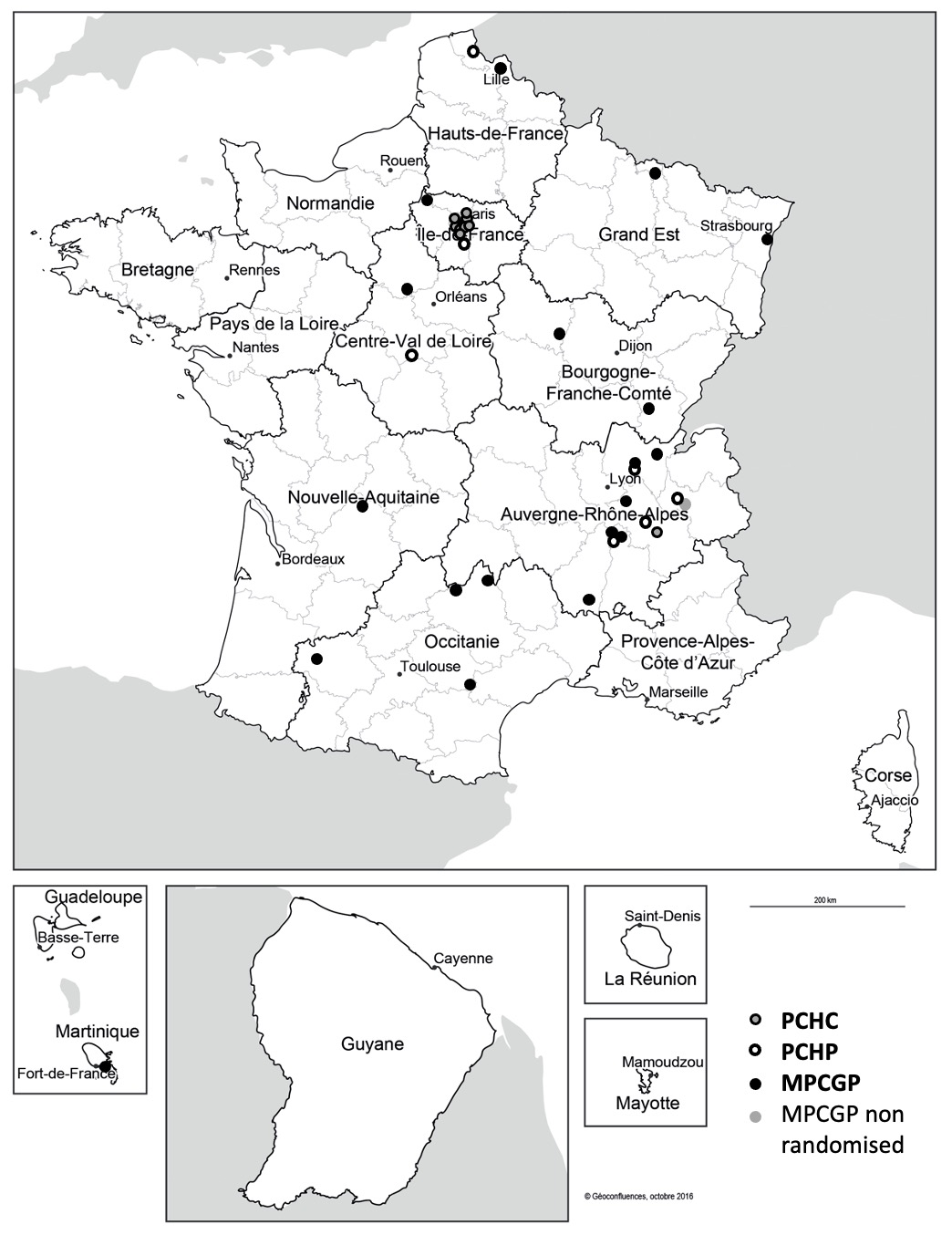

Supplement: Supplementary file 1 — Supplementary Material 1. [file 12875_2024_2476_MOESM1_ESM.docx]
