# Supplementary material for: A multifaceted risk management program to improve the reporting rate of patient safety incidents in primary care: a cluster-randomised controlled trial
Source: BMC Prim Care. 2024 Jul 6;25:244. doi: 10.1186/s12875-024-02476-4 (PMC11227140; doi:10.1186/s12875-024-02476-4)
Supplement: Supplementary file 2 — Supplementary Material 2. [file 12875_2024_2476_MOESM2_ESM.docx]

**Appendix 2: Content (and duration) of the video and of the four interactive training modules**

| 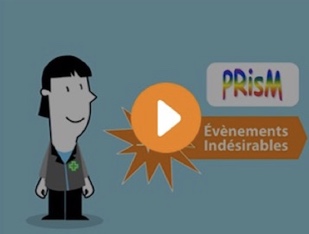 | **Video (3 min): Patient Safety Incident reporting**  **Objectives: Short explanatory video to act on behavior of all professionals** (encourage the detection and reporting of PSI to make professionals good PSI detectors):  -Minimum level of general knowledge: risk management, PSI, PSI analysis...  -Raising awareness and encouraging professionals to detect and report PSI  **Content**:   - Epidemiological data on Patient Safety Incident (PSI) in France - Definition of a PSI in the PRisM study - Awareness on PSIs detection and reporting on the reporting system |
| --- | --- |
| 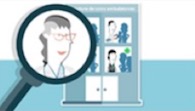 | **Interactive module 1 (14 min): What are EFC and MMR meetings?**  **Objectives:** to know how an Experience Feedback Committee (EFC) and a Morbidity and Mortality Review (MMR) work and to be able to contribute actively in it (for all professionals)  **Content**:   - How an EFC / RMM cycle runs? - Common features and differences between EFC and MMR (PSI choice, participants, different roles…) - Final test : knowledge test and role-playing |
| 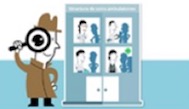 | **Interactive module 2 (11 min): How to investigate a PSI?**  **Objectives:** to be able to build the chronology of a PSI, identify its main cause, its contributing factors and propose corrective measures (Role: “PSI analysis pilot”).  **Content**:   - PSI analysis rules - Restore the chronology of PSI - Identify the contributing factors - Propose appropriate corrective measures - Final test : knowledge test and role-playing |
| 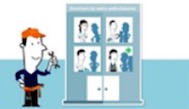 | **Interactive module 3 (10 min): How to manage corrective actions?**  **Objectives**: to be able to implement and monitor corrective measures over time (Role: “pilot of corrective measures”).  **Content**:   - Implement corrective measures - Regularly monitor them and propose their reassessment if necessary - Communicate on the follow-up of these actions during meetings - Final test : knowledge test and role-playing |
| 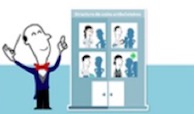 | **Interactive module 4 (13 min): How to run a series of EFC and MMR meetings?**  **Objectives**: to be able to manage EFC and MMR meetings within the facility (Role: “Risk management advisor”)  **Content**:   - Conditions for a successful meeting cycle - Organize, lead, manage meetings and communicate results - Final test : knowledge test and role-playing |
