# Supplementary material for: A multifaceted risk management program to improve the reporting rate of patient safety incidents in primary care: a cluster-randomised controlled trial
Source: BMC Prim Care. 2024 Jul 6;25:244. doi: 10.1186/s12875-024-02476-4 (PMC11227140; doi:10.1186/s12875-024-02476-4)
Supplement: Supplementary file 3 — Supplementary Material 3. [file 12875_2024_2476_MOESM3_ESM.docx]

| **Appendix 3: Main dimensions and sub-levels of CADYA (Categorization of Errors in Primary Care)** | | | |
| --- | --- | --- | --- |
| **MAIN DIMENSIONS** | **SUB-LEVEL 1** | **SUB-LEVEL 2** | **EXPLORED ASPECT** |
| **ENVIRONMENTAL FACTORS** | PATIENT’S SOCIAL CONTEXT |  | Patient’s social situation and life context (broadly defined) |
|  | BACKGROUND OF CARE | UNPLANNED CONSULTATION | Emergency or unscheduled request |
|  |  | PLACE OF CARE | Patient seen in office, at home or elsewhere (care facilities) |
|  |  | WORKLOAD MANAGEMENT | Delay, lack of available appointment or overload |
|  | DISRUPTIVE ELEMENT |  | Physical parasite (telephone, interruption by third party) |
|  | HEALTH SYSTEM | HEALTHCARE SERVICE | Lack of providers or inadequate offer |
|  |  | FINANCIAL OR ADMINISTRATIVE ISSUE | Lack of social coverage, all financial aspects delaying care |
| **HUMAN FACTORS** | LINKED TO THE PATIENT  LINKED TO THE PROVIDER  LINKED TO OTHER PROVIDERS  LINKED TO A THIRD PARTY |  | Inappropriate feelings in the relationship (e.g., lack of empathy)  Behavioural problems (violence, anger)  Fatigue or stress (altering individual performance)  Beliefs and representations (cultural, religious, or ethical) |
| **TECHNICAL FACTORS** | EQUIPMENT | FAILURE, MALFUNCTION, UNAVAILABILITY | All defects connected with the equipment |
|  |  | INCORRECT USE | Non-compliance with regular use of the equipment (knowingly or not) |
|  | INFORMATION SYSTEM | INCORRECT OR MISSING DATA | False or missing information (e.g., reference to an allergy) |
|  |  | FAILURE OF THE COMMUNICATION SYSTEM | Information could not be transmitted in a timely manner (e.g., by fax or email, or mail was not received) |
| **PROCESS OF CARE** | COGNITIVE DIMENSION | LACK OF TRAINING | Fact or knowledge not taught or not memorized |
|  |  | INCORRECT RECALL (AFTER TRAINING) | Failure to recall knowledge or incomplete recall of knowledge |
|  |  | INCORRECT SYNTHESIS | Incorrect decision despite training and reasoning that seems suitable |
|  | CARE PROCEDURE | INAPPROPRIATE OR UNACHIEVED PROCEDURE | All care procedures not achieved or partially achieved |
|  |  | LACK OF PROTOCOL | Faulty, not actualized, or missing protocol |
|  | CARE COORDINATION | COMMUNICATION FAILURE | Miscommunication - other than a failure of the information system |
|  |  | LACK OF (OR INCORRECT) MONITORING | Failure in setting a follow-up or unsuitable monitoring |
|  |  | LACK OF RESPONSE AFTER FEEDBACK | Follow-up not considered or lack of decision after feedback |
